# Excised Femoral Heads in Hip Fracture Patients: Is Osteoporosis Worse Than Cancer?

**DOI:** 10.7759/cureus.6455

**Published:** 2019-12-23

**Authors:** Omer Salar, Sebastien Crosswell, Rafia Ghani, Prasad Rao, Carl Meyer, Stuart Hay, David Ford, Charles Mangham, Paul Cool

**Affiliations:** 1 Orthopaedics, Russell's Hall Hospital, Dudley, GBR; 2 Orthopaedics, Royal Shrewsbury Hospitals National Health Service Foundation Trust, Shrewsbury, GBR; 3 Orthopaedics, Robert Jones and Agnes Hunt Orthopaedic Hospital National Health Service Foundation Trust, Oswestry, GBR

**Keywords:** orthopaedics, osteoporosis, malignancy, cancer, orthopaedics, neck of femur, osteoporosis, fracture, malignancy, survival, cancer

## Abstract

Introduction

Annually 80,000 hip fractures are treated at an estimated cost of two billion pounds. The 2011 guidance from the Royal College of Pathologists recommended all specimens where there is fracture through or below the articular surface should be examined to exclude/ identify an underlying cause (pathological fracture). The questions posed in this study are three-fold. Firstly, how does our practice for hip fracture patients comply with the above audit standards? Secondly, what is the prognostic significance of a past medical history of malignancy on survival? Thirdly, is there any other prognostic survival difference attributable to the diagnosis concluded from the histological analysis of the excised femoral head specimens?

Methods

A retrospective analysis of all hip fractures receiving joint arthroplasty was undertaken between January 2011 and March 2014. Mortality was recorded for a minimum follow-up of 30 months post-operatively. Each excised femoral head was histologically examined by a single consultant histopathologist, and all pre-operative X-rays were reviewed by a consultant radiologist. Histological diagnoses were recorded, and statistical analysis including Kaplan-Meier survival was performed.

Results

A total of 327 consecutive fractures were identified. Out of 187 specimens sent for analysis, only two revealed metastatic deposits in patients with known disseminated malignancy. A previous medical history of malignancy did not confer a significant increase in mortality over a five-year postoperative period (*p *= 0.42). A histological diagnosis of osteoporosis significantly increased mortality over a five-year postoperative period (*p *= 0.004). A comparative analysis found that patients with a histological diagnosis of osteoporosis had the poorest survival.

Conclusion

A histological femoral head analysis may diagnose previously undiagnosed osteoporosis, allowing the clinician to intervene in a disease process, which if left untreated, can lead to a significant increase in mortality.

## Introduction

A total of 80,000 hip fractures are treated annually in the United Kingdom at an estimated cost of two billion pounds in direct healthcare expenditure alone [1-3]. According to the 2014 National Hip Fracture Database (NHFD), 90% of patients who sustained a displaced intracapsular fracture in the United Kingdom received a hemiarthroplasty and 9.9% were treated by a total hip replacement. Both procedures involve intra-operative removal of the femoral head [3]. There is no consensus in either the United Kingdom or the United States with regard to whether the femoral head and associated tissue should be routinely removed during hip fracture surgery and sent for histological examination [4].

Dichotomous opinions exist regarding the value of sending a removed specimen for histological and pathological analysis. Individuals in support of this practice argue that analysis is always beneficial for all types of tissue since the information provided may inform patient management and may improve standards for clinical service through quality-control protocols [4-5]. Antagonists of this practice maintain that the information gained by histological examination of some tissue specimen, including tissue excised during joint replacement, is so rarely beneficial that examination is not warranted and is indeed a costly undertaking [4]. Many surgeons elect to only sent specimens from patients with a suspicious history of a potential pathological fracture for analysis. This may aid in the identification and future management of fractures sustained secondary to metastatic disease, fractures sustained as a result of metastatic disease from an unknown primary source and indeed primary bone tumours or pathologies [6].

The Royal College of Pathologists for the United Kingdom have published guidance regarding articular surface specimen in the tissue pathways for bone and soft tissue pathology [7]. The College advocates that a specimen should be sent for analysis in all hip fracture cases where a fracture is present through or below the articular surface to exclude an underlying pathological cause for the fracture. The recommendation also states that a synovial specimen should be sent for the microspcopic examination in addition to a bone or cartilage specimen.

This study aims to address three main queries: First, how does our institution’s practice for hip fracture patients comply with the standards set by the Royal College of Pathologists? Second, what is the prognostic significance of a past medical history of malignancy on the survival of hip fracture patients? Finally, is there any other prognostic survival difference attributable to the diagnosis concluded from the histological analysis of excised femoral head specimens?

## Materials and methods

A retrospective analysis of all hip fractures treated by joint arthroplasty at Robert Jones and Agnes Hunt Orthopaedic and District Hospital NHS Trust between January 2011 and March 2014 was undertaken. Patients were identified from a prospectively maintained trauma database at a single institution. Baseline patient demographics were collated, including a past or current medical history of malignancy. Operative details including the type of operation performed were recorded. Mortality was recorded for a minimum follow-up of 30 months post-operatively. The database was continuously updated using national and primary care information. A Consultant Radiologist reviewed pre- and post-operative femur and pelvic radiographs for each patient. Any suggestion of a pathological process was recorded.

Excised femoral heads were treated and prepared for histological examination uniformly by our Histopathology Department. The excised femoral heads were sliced with a band saw (average nine bone blocks). Radiographic examination of a slice through the foveal region, 0.3 to 0.5 mm thick, was undertaken. Bone blocks were decalcified in formic acid over several days as per Departmental policy. Once the decalcification process was complete, the bone blocks were embedded in paraffin wax. Paraffin wax bone blocks were rough-cut on microtome, chilled, cut into sections, mounted on slides and heated to 60 degrees celsius for 20 minutes. The specimens were stained with haematoxylin and eosin and examined using an Olympus BH2 light microscope. Pathological features in the radiographs and sections were recorded, including histological diagnosis. A Professor of Histopathology examined and reported on all specimens.

Statistical analysis was conducted using R version 3.5.2 with a *p*-value of <0.05 considered significant. Descriptive statistics were used for baseline demographics. Kaplan-Meier survival analysis was also performed.

## Results

Baseline results

A total of 327 consecutive hip fractures were identified in the study period. Baseline demographics and the incidence of former or current cases of malignancy are shown in Tables 1 and 2, respectively. In total, 23 patients (7%) underwent reconstruction with a total hip replacement and 304 patients (93%) were treated by hemiarthroplasty, and 158 patients (48%) received a cemented prosthesis and 169 (52%) received an uncemented prosthesis.

**Table 1:** Patients baseline demographics included in study (n = 327)

Gender	Age Group (years)			
	61-70	71-80	81-90	91-100
Male	4 (1%)	15 (5%)	42 (13%)	13 (4%)
Female	9 (3%)	57(17%)	134 (41%)	53 (16%)

**Table 2:** Types of current or past malignancy found in patients from their past medical history or clinical notes RCC, renal cell carcinoma; CLL, chronic lymphocytic leukemia; BCC, basal cell carcinoma

Past malignancy	No of Patients (n = 44)
Breast	10
Colon	9
Prostate	4
Lung	3
Oral SCC	2
RCC	2
Meningioma	2
CLL	2
Skin BCC	2
Rectal	2
Gastric	1
Bladder	1
Ovarian	1
Haemangioma	1
Melanoma	1
Myeloma	1

**Figure 1:**
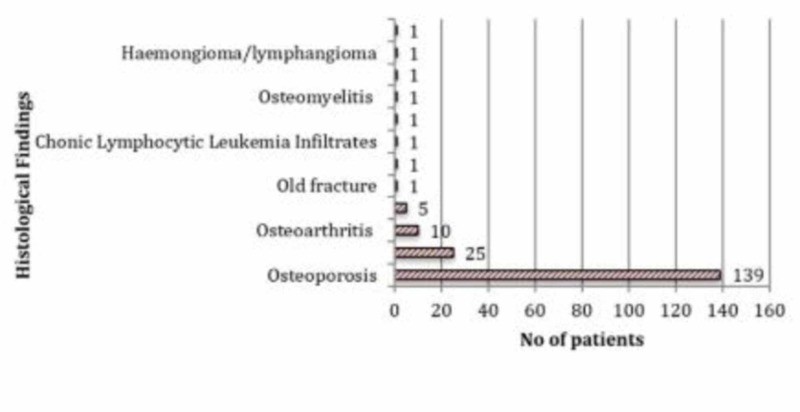
Graph showing reported results of analysis of excised femoral heads following histopathological examination (n = 187)

Femoral heads were excised from 187 patients (57%) and sent for histopathological examination (Figure 1).

**Figure 2:**
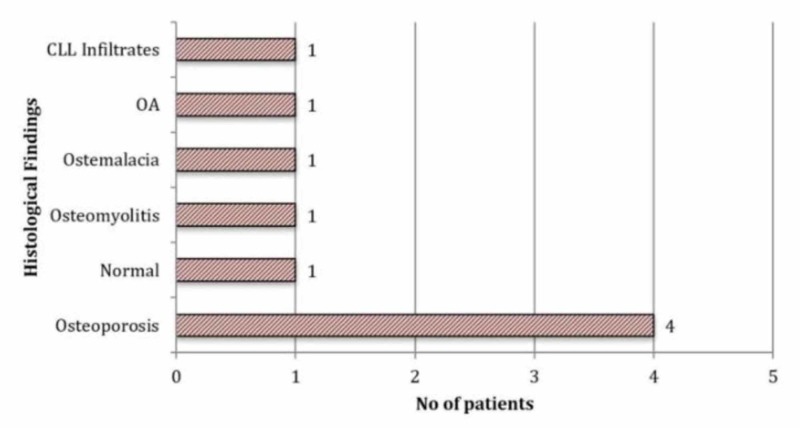
Graph showing femoral head histology results of patients who had suspicious features reported on plain radiographs (n = 9)

Of the 187 femoral heads sent for analysis, only two revealed metastatic deposits. Both specimens were taken from patients with a pre-operative diagnosis of active malignancy, one with known chronic lymphocytic leukaemia (CLL) and one with metastatic prostate cancer. In patients with known or previous malignancy, two out of 31 (6%) had an indication of malignant disease on histological analysis of their femoral head. Eleven radiographs were reported as suspicious for a pathological process, and nine of these patients (82%) had their femoral heads removed and sent for examination. Osteoporosis accounted for the majority of the diagnoses in this group (44%; Figure 2). Overall mortality was 16% at 6 months and 20% at 12 months.

 

 

Survival analysis for patients with a past medical history of malignancy 

**Figure 3:**
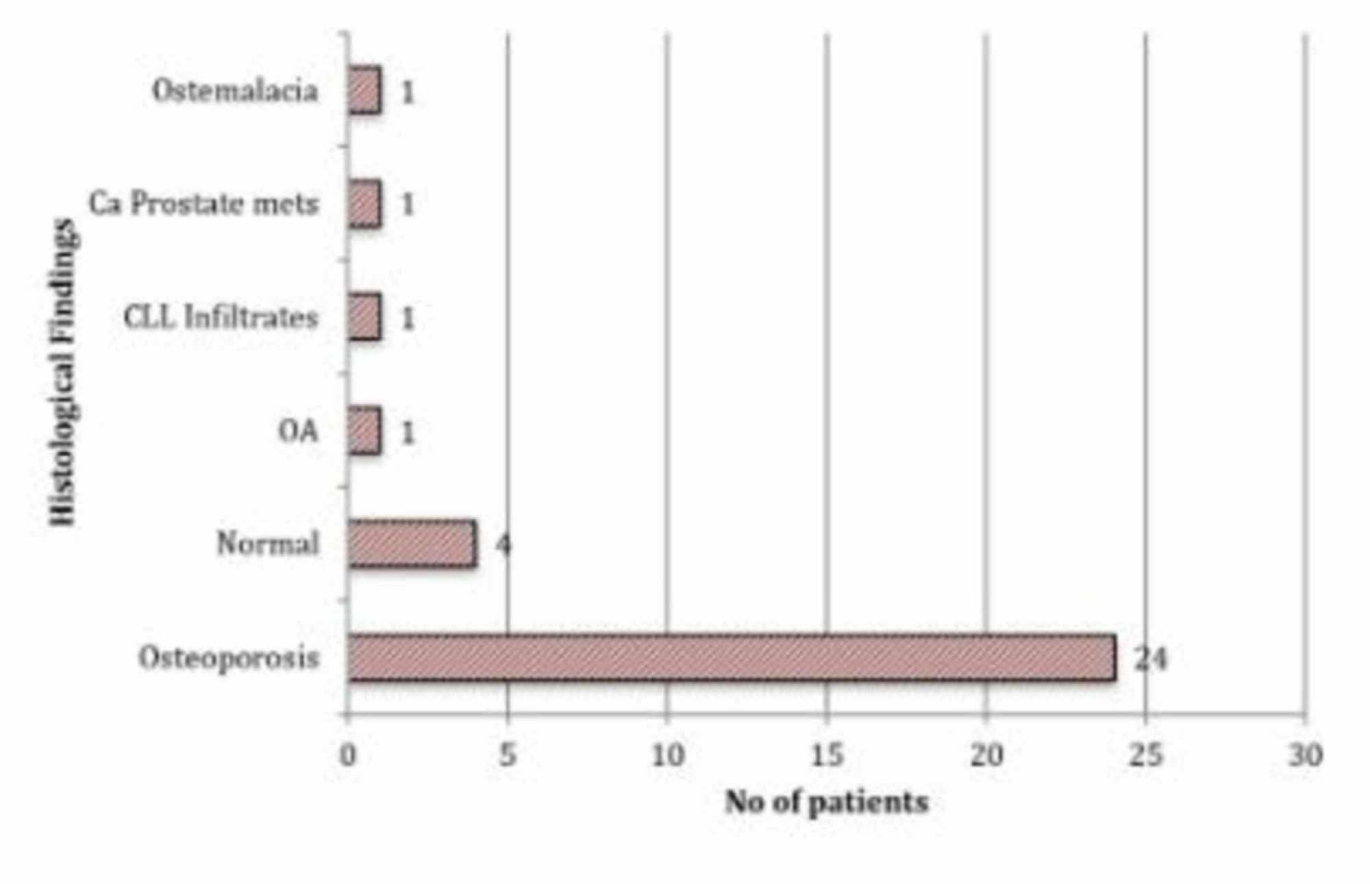
Graph showing femoral head histology results of patient with past or current history of malignancy (n= 32)

In all, 43 (13%) patients in our cohort had a current or past medical history of malignancy. Breast, colon, and prostate malignancies were amongst the most prevalent diagnoses, accounting for 23 patients (54%) in this group (Table 2). Thirty-two of the 43 (74%) patients with a past or current history of malignancy had their excised femoral heads sent for histopathological examination (Figure 3).

**Figure 4:**
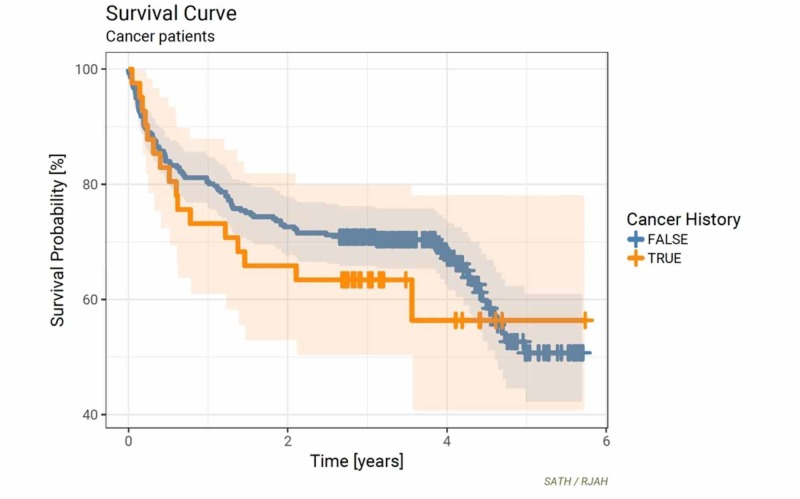
Kaplan-Meier curves for previous malignancy vs all others (p = 0.42)

A diagnosis of osteoporosis was made in 24 (75%) cases and osteoarthritis in one (3%) case. In four (13%) cases, histological examination revealed normal bone architecture in the context of an acute traumatic fracture. Kaplan-Meier survival analysis compared 43 patients with a past medical history of malignancy to the rest of the cohort (Figure 4).

Overall, there was no statistically significant difference in mortality over a five-year post-operative period (*p* =0.42) between the two groups. However, at 12 and 36 months, there was an approximate 5% to 10% negative survival difference for specimens derived from patients with a past medical history of malignancy compared to the rest of the cohort. Conversely, at 60 months, a past medical history of malignancy conferred a similar 5-10% increase in survival. 

Survival Analyses for patients with a histological diagnosis of osteoporosis 

**Figure 5:**
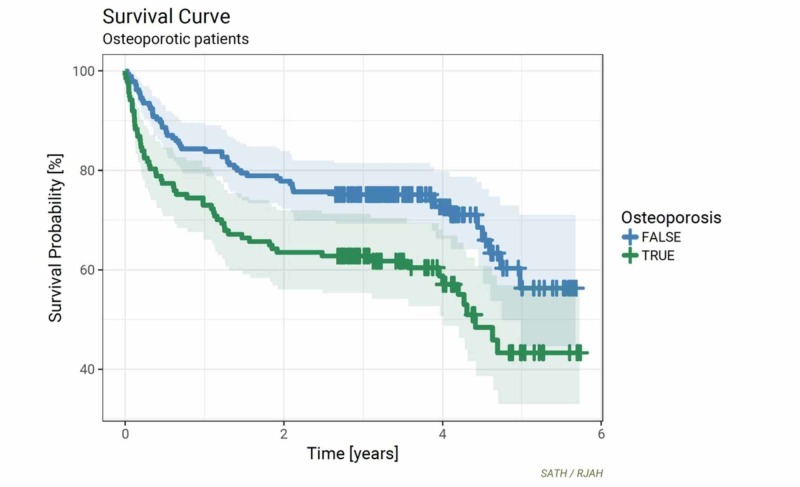
Kaplan-Meier curves for osteoporosis vs all others (p = 0.004)

Osteoporosis was diagnosed on 74% of the specimens sent for histological analysis, which corresponded to a new diagnosis of osteoporosis being made in 139 (43%) of patients (Figure 1). A diagnosis of osteoporosis on histopathology assessment conferred a significantly reduced survival rate when compared to the rest of the cohort over a five-year post-operative period (*p *=0.004). The difference in survival was most noticeable at 36 months and 60 months post-operatively where a histological diagnosis of osteoporosis conferred an approximate 15% negative survival difference compared to the rest of the cohort (Figure 5).

## Discussion

Femoral heads were sent for histological analysis in just 57% of cases, highlighting significant inter-surgeon variability with regard to this practice. Of the 187 femoral heads that were analysed, only two (1%) revealed metastatic disease in patients with a known diagnosis of malignancy. More successful contemporary screening programs show significant detection rates. For example, breast cancer screening detects six new malignancies per 1904 screened women and flexible sigmoidoscopy has been reported to detect five colorectal cancers per 489 people screened [8].

There is a limited amount of published literature on this topic, the majority of which focuses on transmissible disease screening in total hip arthroplasty for elective surgery for osteoarthritis. These studies maintain that the practice of histological examination and the potential diagnostic information that may be gained from this process may only occasionally affect patient management [4-9]. Conversely, a retrospective review of 6161 femoral head histology reports from total hip arthroplasty or specimens donated at death performed by Mackie et al. found 19 femoral heads, which contained neoplastic changes, making the rate of malignancy detection one per 770 femoral heads [10].

Although no new malignancies were identified in our study group through histological femoral head analysis, the process was instrumental in diagnosing 74% of our hip fracture patients with osteoporosis. Survival analysis clearly demonstrated that a diagnosis of osteoporosis on histology conferred a significant negative prognostic implication at all time points across the 60-month follow-up period. This was most notable at the 36- and 60-month time points, where patients with a diagnosis had a 15% increase in mortality compared to the rest of the cohort. Osteoporotic fractures are associated with higher rates of hospitalization, a decrease in functional status and independence and higher mortality rates [11-15]. Despite this, notable statistics data from North America suggests that only 20% of patients who are diagnosed with osteoporosis actually receive treatment for the condition [16-17].

The reasons for this are clearly multi-factorial and lie with both the patient as well as the surgeon or treating physician. Surgeon or physician factors cited in contemporary literature include individuals feeling undertrained to treat osteoporosis, a lack of appropriate knowledge to treat the condition correctly and perceived side effects or doubts over the effectiveness of treatment [18-20]. Patient factors cited in existing literature include a lack of appreciation of the link between osteoporosis and the fracture that they have sustained [21].

The index surgeon and/or physician who is involved in the management of the fragility fracture is central to ensuring that an identified case of osteoporosis is treated and it is therefore incumbent upon them to instigate this process [22]. Current data clearly demonstrates that the treatment of osteoporosis from the time of index fracture significantly reduces secondary re-fracture rates [23-24]. The diagnosis of osteoporosis on histology empowers the lead clinician to instigate treatment and ensure that the patient receives continuous support and management of their condition through effective communication with primary services.

Interestingly, comparative survival analysis demonstrated that patients who had an existing or previous malignancy had better survival rates than patients with a histological diagnosis of osteoporosis. This finding further highlights the importance of diagnosis, management and care of patients who sustain a fragility fracture. What is difficult to ascertain is if these patients had previous opportunities missed to highlight the possibility of fragility fractures prior to their hip fracture. Nonetheless, it is well known that the uptake of treatment in this group remains poor, and this study emphasizes the grave consequences of such a trend.

The NHFD was established in an effort to improve morbidity and mortality in hip fracture patients. One of its hallmark recommendations is that every hip fracture patient is reviewed by an orthogeriartrician within 72 hours of admission. Consideration of bone protection requirements is central to this assessment. Despite these guidelines and the improvement in mortality demonstrated by the introduction of NHFD guidelines, compliance with this particular recommendation remains variable across the United Kingdom. The authors of this study would advocate that a new diagnosis of osteoporosis should be urgently highlighted to primary/ community services for bone health. Consideration should be given to the creation of a fast track system to ensure that these patients receive treatment for their osteoporosis as well as a thorough assessment and management plan for associated general health ailments that may contribute to increased morbidity and mortality if left untreated.

The increased survival rates demonstrated in patients with a current or past history of malignancy could be attributed to recent improvements in modern cancer treatments. The National Office for Statistics published data which revealed that “5-year net survival estimates for 2010 to 2014 were up to 2.3% higher than the corresponding figure for 2009 to 2013 in men (thyroid cancer increasing from 80.5% to 82.8%) and up to 2.1% higher for women (renal cancer increasing from 60.8% to 62.9%)” [25].

With cancer survival increasing as a result of early identification and improved treatment response, the role of femoral head histology to identify metastatic disease appears to be limited. However, despite only 57% adherence to the National Guidelines, our study clearly demonstrated an increased mortality rate in patients who have a histological diagnosis of osteoporosis. As such, perhaps guidance should be changed to identify osteoporosis based on histological evidence. Given the cost of bisphosphonates and osteoporotic medical management, with their own set of complications, basing medical management on histological diagnosis could potentially be more cost-effective. Identification of osteoporosis and treatment of this condition at this stage could result in reduced risk of future fractures and improved overall mortality.

Limitations

The data is limited in its retrospective nature and that it has been obtained at a single institution. This may limit its extrapolation and national application. The data collected and analysed in this paper have been collected retrospectively compared to the national data on cancer rates and survival which is collected prospectively from multiple sources. Bias may have been introduced by the limited number of excised femoral heads submitted for histological review.

## Conclusions

Our study found that individuals with histologically confirmed osteoporosis have a significantly worse prognosis compared to other patients. We therefore recommend histological analysis of all excised femoral heads to help diagnose osteoporosis. We recommend treating osteoporosis when it is first identified to reduce morbidity and mortality in this patient group. It is our belief that it is the responsibility of the index physician and/or surgeon to initiate treatment and further investigation of patients who are diagnosed with osteoporosis based on histological analysis. In-hospital management should be supported by continuous input from primary care services.
